# Cardiac metastasis in uterine cervical cancer: A systematic review and case study

**DOI:** 10.1007/s00066-024-02274-y

**Published:** 2024-09-17

**Authors:** I.-M. Simek, A. Sturdza, J. Knoth, A Spannbauer, J. Bergler-Klein, M. Vögele-Kadletz, J. Widder, M. P. Schmid

**Affiliations:** 1 Department of Radiation Oncology, Comprehensive Cancer Center, Medical University of Vienna, General Hospital of Vienna, Währinger Gürtel 18–20, 1090 Vienna, Austria; 2 Department of Cardiology, Medical University of Vienna, General Hospital of Vienna, Vienna, Austria; 3 Department of Cardiac Surgery, Medical University of Vienna, General Hospital of Vienna, Vienna, Austria

**Keywords:** Cardiac mass, Dyspnea, Arrythmia, Cardiac surgery, Thrombosis

## Abstract

**Purpose:**

Cardiac metastasis from cervical cancer is rare and only scarcely documented. We aim to present a new case and systematically summarize the available literature.

**Materials and methods:**

PubMed, Scopus, Web of Science, Central, and ClinicalTrials.gov were systematically searched following the PRISMA (Preferred Reporting Items for Systematic Reviews and Meta-Analyses) criteria. Results were screened via title, abstract, and full text. Additionally, the reference lists of all papers chosen for the review were screened.

**Results:**

Eighty-one papers were identified, describing 86 cases in total. Cardiac metastasis occurred at all stages of cervical cancer and in all age groups. Median time from initial diagnosis to diagnosis of cardiac metastasis was 12 months. Patients mainly complained of dyspnea and chest pain, 60.8% had pathologic ECG (electrocardiographic) findings. The cardiac mass was most frequently detected by transthoracic echography. The most common tumor histology was squamous cell carcinoma. Chemotherapy and surgical interventions were the main treatment modalities. Median survival after diagnosis of cardiac metastasis was 3 months.

**Conclusion:**

This largest review on cardiac metastases from cervical cancer confirmed the heart as a very infrequent site of metastasis. There are < 100 cases described in the literature, with very poor prognosis and undefined clinical management.

## Introduction

Cardiac metastasis is exceptionally uncommon in cervical cancer. The clinical presentation, optimal management, and course of disease are therefore ill defined. To identify eventual treatment patterns, we conducted a systematic review to assess the most common clinical characteristics, diagnostic findings, and clinical outcome of cardiac metastasis in cervical cancer. The aim of this study is primarily to present the results of the systematic review and, secondly, to present a case report of a patient with cardiac metastasis from cervical cancer.

## Case report

A 71-year-old patient with locally advanced cervical cancer was referred to the Department of Radiation Oncology of the Medical University of Vienna. Clinical examination and pelvic MRI (Magnetic Resonance Imaging) showed a 5-cm tumor in the uterine cervix with parametrial infiltration. PET-CT (Positron Emission Tomography-Computed Tomography scan) revealed multiple suspicious pelvic and paraaortic lymph nodes but no evidence of distant metastasis (FIGO stage IIIC2; International Federation of Gynecology and Obstretrics). Histology was a highly dysplastic p16-positive squamous cell carcinoma. The multidisciplinary tumor board recommended primary radiochemotherapy and image-guided adaptive brachytherapy [1].

The patient received external beam radiotherapy (EBRT) to 45 Gy with 1.8 Gy per fraction delivered to the pelvis and the paraaortic lymph node regions and a simultaneous integrated boost to suspicious lymph nodes (57 Gy with 2.28 Gy per fraction). Concomitant to EBRT, five cycles of cisplatin 40 mg/m^2^ were administered.

**Fig. 1:**
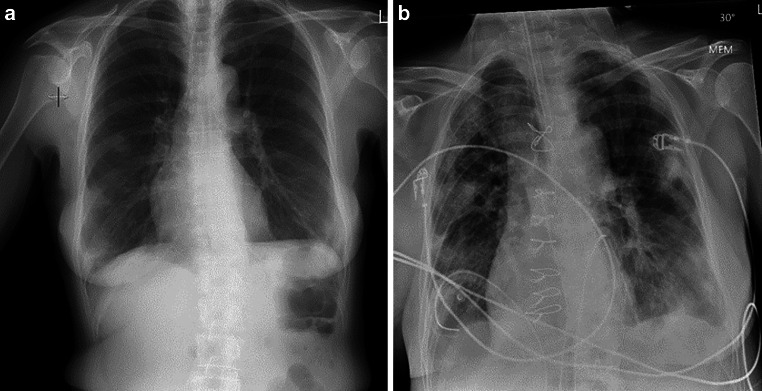
**a** Routine X‑ray before brachytherapy showing patchy alterations in both lungs; **b** X-ray one day after cardiac surgery

A chest X‑ray performed as part of the routine work-up before brachytherapy (Fig. 1) showed infiltrations suspicious for pneumonia.

**Fig. 2:**
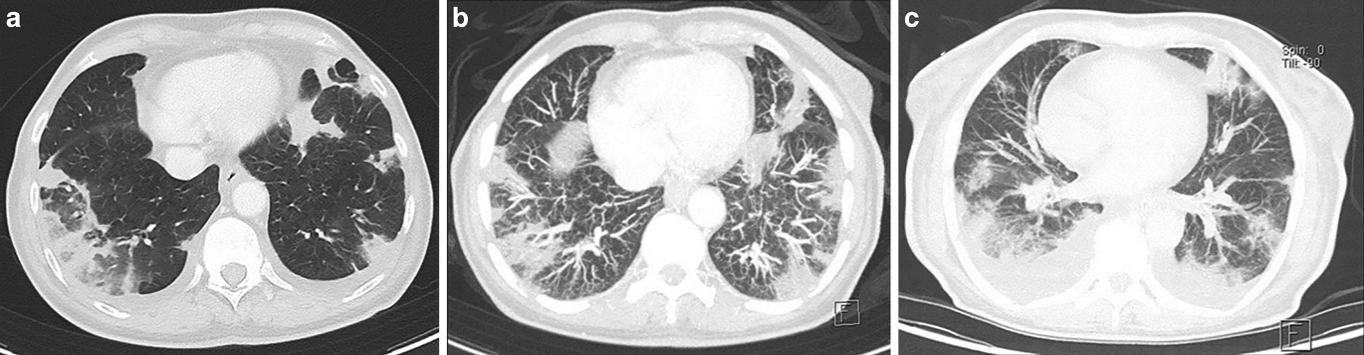
**a** First CT scan after initial diagnosis of a cardiac mass; **b** second CT scan about 2 weeks later; **c** third CT scan another 2 weeks later

C‑reactive protein levels were elevated; however, the patient did not present any respiratory symptoms or fever. She received antibiotics and brachytherapy was postponed. A follow-up chest X‑ray after 6 days indicated progressive infiltrations, and subsequently a thoracic CT (Fig. 2a) was performed, showing patchy alterations in both lungs that were interpreted as organizing pneumonia; malignant metastatic embolism was indicated as a possible differential diagnosis in the report.

**Fig. 3:**
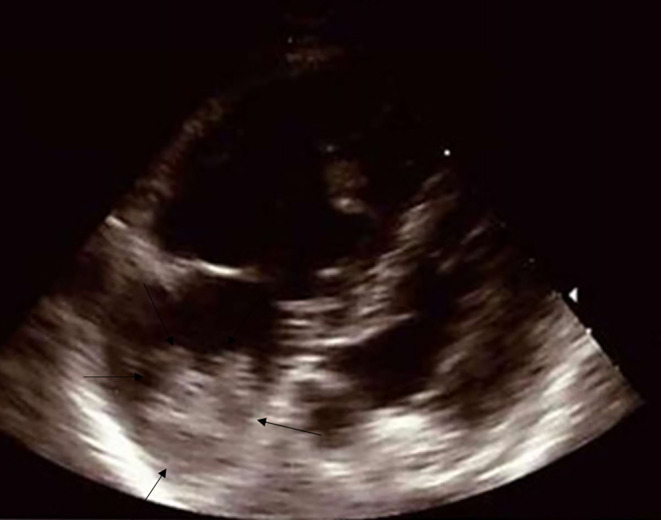
Transthoracic echography showing a floating structure in the right atrium

Transthoracic sonography was repeatedly performed, which showed a floating structure in the right ventricle near the tricuspid valve (18 × 19 mm), as well as a highly mobile structure in the right atrium (50 × 20 mm). Both structures were suspicious of a thrombus (Fig. 3).

As differential diagnoses, fresh vegetation and malignancy were discussed. Moderate right ventricular hypertrophy with apical enlargement and abnormal septal motion due to increased pulmonary systolic pressure (systolic PAP of 94 mm Hg, TI velocity 4.6 m/s) was observed, in accordance with pulmonary embolism. Moderate tricuspid regurgitation and mild pericardial effusion in front of the right ventricle and atrium was also present [2]. Troponin was slightly elevated, at 31 pg/ml.

**Fig. 4:**
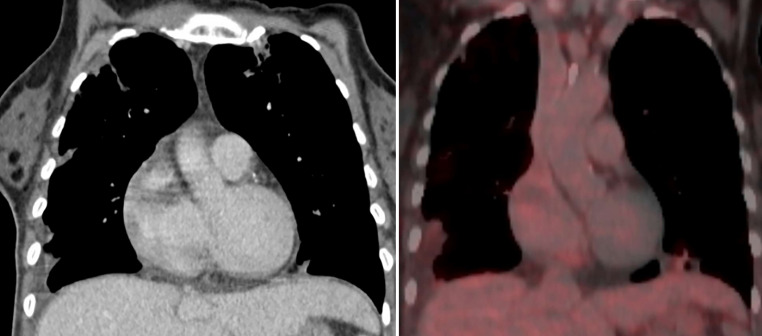
PET-CT (positron emission tomography-computed tomography) scan showing a 20-mm mass in the right ventricle

A PET-CT (positron emission tomography-computed tomography) scan was performed that showed no evidence of distant metastasis and no clear FDG (fluorodeoxyglucose) enhancement of the 20-mm cardiac mass, but progressing pulmonary embolism was implied and cardiac MRI was recommended (Fig. 4). The patient was treated with therapeutic doses of low-molecular heparin.

In the meantime, the general condition of the patient deteriorated, with increasing dyspnea, tachycardia (~110 bpm), weakness, and hyperglycemia.

After multidisciplinary discussion—taking the PET-CT scan and progressing clinical symptoms into account—the patient was offered open-heart surgery for removal of the unclear mass instead of further diagnostic procedures. Intraoperatively, the right ventricle appeared extremely hypertrophic. In the right atrium and ventricle, a jellylike mass was found, which was attached to the trabeculae but did not seem to infiltrate them. Frozen sections indicated squamous cell carcinoma.

During surgery, pulmonary effusions were drained. The pulmonary tissue in the affected areas almost resembled cartilage.

During surgery, the patient’s condition deteriorated, and she was transferred to the intensive care unit, where she died 2 days later due to cardiorespiratory failure. The final histology confirmed HPV-positive (human papillomavirus) cardiac squamous cell carcinoma metastasis from cervical cancer. No autopsy was performed due to the wish of the family. The multiple pulmonary emboli were interpreted as malignant metastatic embolization.

## Materials and methods

### Database search

**Fig. 5:**
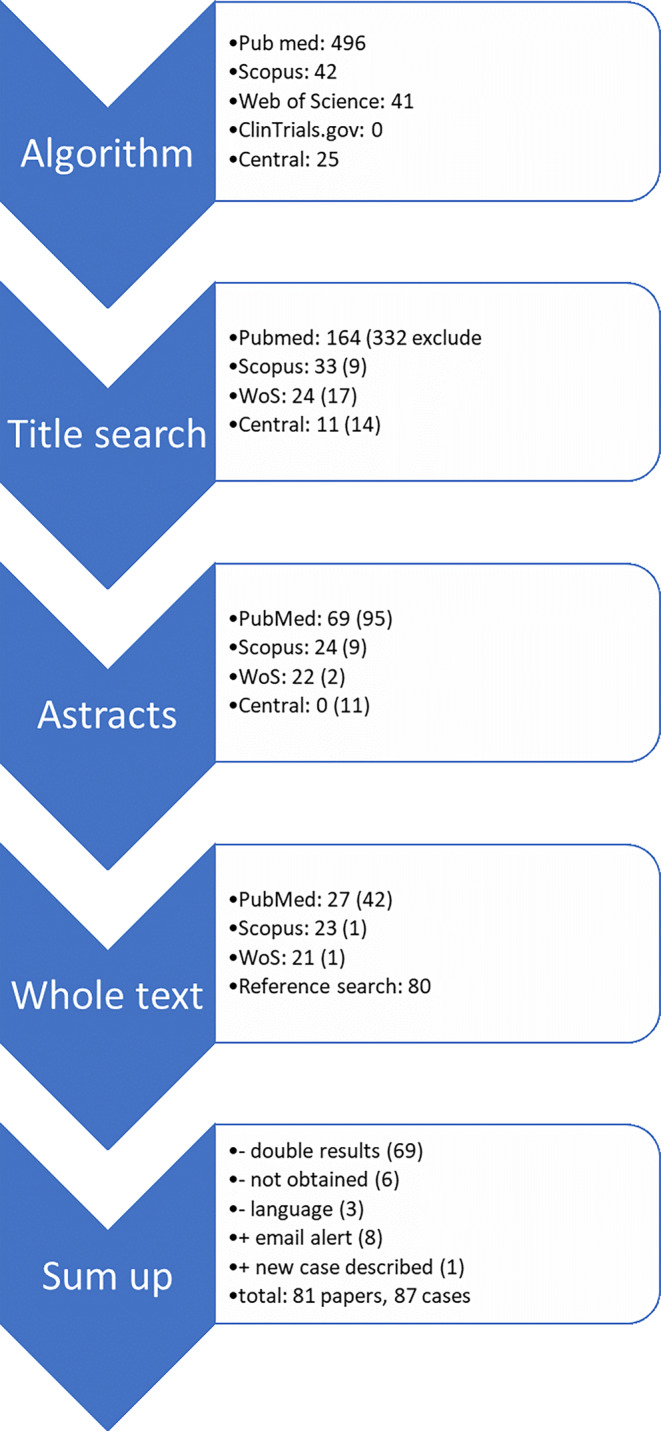
PRISMA (Prefered Reporting Items for Systematic reviews and Meta-Analyses) diagram of the research process

A systematic literature search employing PRISMA (Fig. 5) guidelines focused on case reports about cardiac metastases from cervical cancer [3]. PubMed was queried using the following search terms: (((*card* OR heart) AND metast*) AND ((cervic* OR uter*) AND cancer)); Scopus, using (“cardiac metastasis” AND “cervical cancer”), Web of Science with ((TS = (uter* AND cervi*)) AND (TS = (card* AND metast*))), CENTRAL with (Disease: cervical cancer/cervix cancer/cervix dysplasia/cervical dysplasia/; Additional terms: card* metast*), and ClinicalTrials.gov via “Disease: cervical cancer/cervix cancer/cervix dysplasia/cervical dysplasia; Additional terms: card* metast*.” Searches were conducted in December 2019, and an e‑mail alert was created for PubMed and Web of Science that was followed until May 2022. Based on the search results, a title search was conducted, followed by an abstract and a full text search. The search was supplemented by cross-referencing the cited references of relevant papers.

### Selection criteria

No prospective studies but rather only case reports, case series, and case reports with additional literature reviews could be identified. Papers were excluded if they did not describe female human patients with a tumor of the uterine cervix metastasizing to the heart. Three papers had to be excluded because of language issues (Japanese, Hungarian, Serbian), and six papers could not be obtained. Seven additional cases were retrieved via the email alert. After removing duplicates, we ended up with 81 papers describing 86 cases of cardiac metastasis in cervical cancer. Additionally, we included our newly described case into the analysis.

## Results

In total, 87 cases of patients with cardiac metastasis from cervical cancer were analyzed and compiled (see the supplement).

Median age at diagnosis of cardiac metastasis was 47 years (21–81 years). Tumor stage at initial diagnosis was FIGO stage I in 16 cases (18.4%), II in 19 (21.8%), III in 21 (24.1%), IV in 10 (11.5%), and unknown in 21 (24.1%) patients; 81/87 (93.1%) had squamous cell carcinoma of the cervix, 3/87 (3.4%) had adenocarcinoma, 2/87 (2.3%) had a neuroendocrine tumor (NET), and 1/87 (1.1%) had malignant melanoma. Treatment for the primary tumor was heterogeneous, including surgery, chemotherapy, radiotherapy, and all possible combinations of those three options. In 8/87 (9.2%) cases, initial therapy for the primary tumor was not stated.

Median time from initial diagnosis to diagnosis of cardiac metastasis was 12 months (ranging from no time to more than 10 years). Dyspnea (55/80; 68.8%), chest pain (25/80; 31.3%), deteriorating general condition (9/80; 11.3%), and arrhythmias (9/80; 11.3%) were the main reported symptoms due to cardiac metastasis, whilst 7/80 (8.8%) patients had no symptoms. ECG findings were reported in 51 cases and were pathologic in 31/51 (60.8%) patients, showing tachycardia (25/31; 80.6%), altered t‑waves (17/31; 54.8%), or low voltage (9/31; 29%). Changes occurred mostly in the right chest leads (10/31; 32.3%), with 5/31 (16.1%) patients having a right bundle branch block. Further diagnostic modalities included transthoracic echography with a visible cardiac mass in 50/68 (73.5%) patients, CT (25/46; 54.3%), MRI (14/18; 77.8%), and PET-CT (15/21; 71.4). In 43/86 (50%) cases the cardiac metastasis was located in the right ventricle and in 17/86 (19.8%) in the right atrium (Table 1); 19/86 (22.1%) patients had an additional cardiac effusion, and in one case the location of the cardiac metastasis was not stated. Pulmonary embolism with cardiac metastasis was detected in 29/87 (33.3%) cases. Overall, 44/87 (50.6%) patients had other organ metastases confirmed, and 56/64 (87.5%) women had positive lymph nodes at diagnosis.

**Table 1:** Overview of all data obtained

**Location of cardiac metastasis**				
*Right*	52	*Effusion*	19	
*Left*	2	*Right atrium*	17	
*Both sides*	10	*Right ventricle*	43	
*Ventricle*	42	*Left atrium*	8	
*Atrium*	17	*Left ventricle*	9	
*Both*	8	*Unknown*	1	
**Time to cardiac diagnosis**		**Survival after cardiac diagnosis**		
*<* *1* *month*	5	*<* *1* *month*	14	
*1–3* *months*	3	*1–3* *months*	19	
*>* *3–6* *months*	9	*>* *3–6* *months*	12	
*>* *6–12* *months*	21	> 6–*12* *months*	15	
*12–24* *months*	16	> *2* *months*	9	
*>* *24* *months*	19	*Unknown*	14	
*Unknown*	1	*Postmortem*	9	
*Postmortem*	3	*Median*	3	
*Median*	11	*Average*	5.4	
*Average*	24.6	**RR**		
**Symptoms of cardiac metastasis**		*High*	5	
*None*	7	*Low*	10	
*Constitutional*	9	*Normal*	14	
*Arrhythmia*	9	*Unknown*	58	
*Others*	31	**SpO**_**2**_		
*Dyspnea*	55	*Normal*	3	
*Chest pain*	75	*Low*	10	
**ECG**		*Unknown*	73	
*Tachycardia*	25	**Treatment for cardiac metastasis**		
*Right leads*	10	*None or unknown*		16
*Left leads*	1	*Surgery*		21
*Block*	8	*Pericardiocentesis*		19
*RBBB*^*a*^	5	*Chemotherapy*		31
*High voltage*	1	*Radiotherapy*		8
*Low voltage*	9	–	**RT**	**ChT**
*Hypertrophy*	2	*None*	10	19
*Other*	6	*Cord. diag.*	17	29
*Altered t‑wave*	17	*Cervix*	58	38
*None*	20	*Both*	12	11
*Unknown*	36	*Unknown*	8	8
–	**TTE**	**MRI**	**CT**	**PET**
*Normal*	1	1	4	4
*Pathological*	67	16	42	17
*No mass visible*	17	2	17	2
*Mass visible*	*50*	14	25	15
*Unknown*	*19*	69	41	66

^a^*RBBB* right bundle branch block, *RR* blood pressure, *ECG* electrocardiogram, *SpO2* oxygen saturation, *TTE* transthoracic echocardiogram, *MRI* Magnetic Resonance Imaging, *RT* radiation therapy, *ChT* chemotherapy, *CT* computed tomography, *PET* positron emission tomography-computed tomography scan

The cardiac metastasis was resected in 31/87 (35.6%) patients, 29/87 (33.3%) women underwent chemotherapy, 19/87 (21.8%) received pericardiocentesis or pericardial windowing, and 8/87 (9.2%) patients had radiotherapy. Treatment of cardiac metastasis was not performed or not stated in 16/87 (18.4%) patients. Median survival time after diagnosis of cardiac metastasis was 3 months, with 9/63 (14.3%) patients surviving for more than 1 year and 13/63 (20.6%) patients surviving for less than a month. In 14/87 (16.1%) cases the survival time was not stated and in 10/87 (11.5%) the diagnosis was made postmortem. Of the cases with known survival time (*n* = 63), median survival was 5 months among patients who had cardiac surgery (*n* = 25) versus 2.5 months in those who had no cardiac surgery (*n* = 38; Table 2).

**Table 2:** General overview of all papers reviewed

Paper	Metastasis	Primary	Staging	Age	Survival
[4]	Sev. lov	SCC	IVb	22	6
[5]	RV	SCC	n. r.	64	n. r.
[6]	RV	SCC	IIb	41	5
[7]	Sev. loc	SCC	n. r.	43	n. r.
[8]	Pericardium	SCC	IIIb	52	n. r.
[9]	Pericardium	AdenoCa	n. r.	54	1
[10]	RV	SCC	IIb	43	12
[10]	Sev. loc	SCC	II	51	p. m.
[10]	RA, RV	SCC	n. r.	65	p. m.
[11]	RA, RV	SCC	IVb	42	n. r.
[12]	RA, RV	SCC	IIa2	32	13
[13]	Pericardium	SCC	IIIb	46	n. r.
[14]	RV	SCC	Ib	56	p. m.
[15]	RV	SCC	n. r.	38	≤ 1
[16]	RV	SCC	n. r.	67	≤ 1
[17]	RV	SCC	II	59	≤ 1
[18]	RV	SCC	n. r.	63	6
[19]	RV	SCC	IIIb	37	8
[20]	RA, LA	Melanoma	Ib	73	3
[21]	RV	SCC	IIIb	77	p. m.
[21]	LV	SCC	IIIb	44	p. m.
[21]	?	AdenoCa	IIb	72	≤ 1
[22]	RA	AdenoCa	IIb	60	≤ 1
[23]	RV	SCC	IVb	44	15
[24]	LV	SCC	Ib	43	5
[25]	RV	SCC	Ib	44	8
[26]	RV	SCC	n. r.	75	?
[27]	Pericardium	SCC	Ib	36	9
[28]	RV, RA	SCC	Ib1	58	5
[29]	RV	SCC	IIa	64	≤ 1
[30]	RV	SCC	Ib	49	3
[31]	Pericardium	SCC	Ib	57	5
[32]	RV	SCC	n. r.	49	n. r.
[33]	RV, LV	SCC	IIb	35	3
[34]	RA	SCC	IVb	52	12
[35]	RV	AdenoCa	n. r.	43	n. r.
[36]	Pericardium	SCC	Ib	64	12
[37]	RV	SCC	IIb	28	3
[this case]	RV, RA	SCC	IIIc2	70	≤ 1
[38]	LA	NET	n. r.	43	24
[39]	RV, RA	SCC	IVb	53	1
[39]	RV	SCC	IVb	49	7
[40]	RV, LV	SCC	Ib	36	2
[41]	Pericardium	SCC	IIIb	42	4
[41]	Pericardium	SCC	IIIb	37	10
[42]	Sev. loc	SCC	II	50	≤ 1
[43]	RV	SCC	IIIc1	35	n. r.
[44]	Sev. loc	SCC	Ib2	48	8
[45]	RV	NET	n. r.	37	2
[46]	RA, RV	SCC	IIIb	64	≤ 1
[47]	RA, IVC	SCC	IIIb	57	2
[48]	Pericardium	SCC	IV	51	4
[48]	Pericardium	SCC	IIIb	61	12
[49]	RV	SCC	n. r.	81	≤ 1
[50]	RA, RV	SCC	IIIa	27	≤ 1
[51]	RV	SCC	IVb	60	p. m.
[52]	Pericardium	SCC	IV	55	≤ 1
[53]	RV, RA	SCC	IIIb	54	2
[54]	Pericardium	SCC	IIIb	38	≤ 1
[55]	Pericardium	SCC	IIa	49	10
[56]	RV	SCC	IIb	33	1
[57]	Pericardium	SCC	n. r.	35	1.5
[58]	Pericardium	SCC	IIIb	68	n. r.
[59]	Pericardium	SCC	n. r.	35	5
[60]	RV	SCC	IIIb	68	5
[61]	RA, LA	SCC	IIIb	47	11
[62]	RV	SCC	n. r.	28	≤ 1
[63]	RA	SCC	n. r.	29	p. m.
[64]	RV, LV	SCC	IIb	33	n. r.
[65]	RV	SCC	Ib	28	1
[66]	Sev. loc	SCC	IIa	50	p. m.
[67]	IVS	SCC	IVb	40	45
[68]	RV	SCC	Ib	36	n. r.
[69]	RA	SCC	n. r.	33	n. r.
[70]	RV	SCC	IIb	48	9
[71]	RV	SCC	IIa2	48	6
[72]	RA	SCC	n. r.	60	n. r.
[73]	RA	SCC	IIa	39	8
[74]	RV	SCC	IIIb	78	1
[75]	Pericardium	SCC	Ib1	38	6
[76]	Pericardium	SCC	IIb	39	12
[77]	RV	SCC	n. r.	46	n. r.
[78]	Sev. loc	SCC	n. r.	60	≤ 1
[79]	LA	SCC	IIIb	54	≤ 1
[80]	LV, IVS	SCC	n. r.	45	2
[81]	RA, RV	SCC	Ib	46	1.5
[82]	Sev. loc	SCC	IV	21	p. m.

*RA* right atrium, *RV* right ventricle, *LA* left atrium, *LV* left ventricle, *IVS* interventricular septum, *sev. loc.* several locations, *SCC* squamous cell carcinoma, *NET* neuroendocrine tumour, *AdenoCa* adeno carcinoma, *p.* *m.* post mortem diagnosis; age in years, survival after cardiac diagnosis in months, *n.* *r.* not reported

## Discussion

Cervical cancer is the fourth most frequent cancer in women worldwide [83]. Depending on the stage at diagnosis, curative treatment of primary disease consists of surgery or radiochemotherapy. In patients with locally advanced disease undergoing primary radiochemotherapy, approximately 20% develop distant metastasis during follow-up [1]. In case of distant metastatic disease, chemotherapy is generally recommended. Distant metastases mainly occur in the lungs, mediastinal lymph nodes, bones, peritoneum, and liver. Metastases to the adrenal glands, spleen, or brain are less frequent [84–86].

Cardiac metastasis of cervical cancer is, however, very uncommon, with only a few case reports and small reviews available [67]. In general, cardiac tumors are rare in clinical practice, with metastases to the heart being much more common than primary tumors [87]. Among primary cardiac tumors, sarcomas are more common than carcinomas [88]. The most common primary tumor of the heart is a benign atrial myxoma [87]. In 11,432 autopsies conducted between 1973 and 2004, Butany et al. found 264 cardiac metastases and two primary cardiac tumors. Cardiac metastasis most frequently originated from lung cancer, the bone marrow (leukemia or multiple myeloma), breast, and lymph nodes (lymphoma). Metastases were found most frequently in the pericardium, followed by the myocardium [87]. Endocardiac and intracavital metastases account for only 3–5% of cardiac metastases [89].

The present paper summarizes the findings of 87 patients with cardiac metastases from cervical cancer. Cardiac metastasis occurred in all stages and in all age groups. Median time from initial diagnosis to diagnosis of cardiac metastasis was 11 months, which is the typical timespan for the occurrence of distant metastasis in cervical cancer [85]. Patients mainly presented with general symptoms such as dyspnea and chest pain, and 60.8% (31/51) had pathologic ECG findings. In agreement, Poterucha et al. reported that cardiac metastasis can present with a variety of symptoms including direct effects from the tumor, general symptoms, and symptoms consecutive to distal embolization [90]. Interestingly, our patient was initially asymptomatic despite the presence of multiple pulmonary emboli on X‑ray and CT. A cardiac mass could then be assessed by transthoracic sonography, which is the (first) imaging modality of choice in case of suspicion of a cardiac tumor [91], which is also reflected by the high usage (72%) in our review cohort. Cardiac MRI might be able to distinguish a metastasis or thrombus from an atrial myxoma.

Chemotherapy and surgical interventions were the main treatment modalities in the reviewed cohort. Regarding the very limited median survival (3 months) after diagnosis of cardiac metastasis, aggressive treatment approaches appear questionable and must be carefully scrutinized. There seems to be a trend towards improved survival after cardiac surgery; however, this finding is most likely due to selection and publication bias. Our patient died soon after an open-heart surgical approach, which was chosen due to the assumed absence of distant metastases and missing FDG uptake of the cardiac tumor and the surrounding emboli, which had thus suggested a non-malignant origin of the cardiac mass. We chose to present this case and the corresponding review to underscore the fact that also very rare clinical scenarios need to be critically discussed and considered in the interdisciplinary management of cancer patients.

In conclusion, cardiac metastasis is an exceptionally rare event in cervical cancer, with < 100 cases described in the literature. The prognosis is very poor. Dyspnea, chest pain, pulmonary embolism, tachycardia, and ECG alterations were the most frequently reported symptoms. The optimal treatment strategy for cardiac metastasis in cervical cancer patients remains undefined.
